# Supplementary material for: Sequencing and comparative analysis of three Chlorella genomes provide insights into strain-specific adaptation to wastewater
Source: Sci Rep. 2019 Jul 2;9:9514. doi: 10.1038/s41598-019-45511-6 (PMC6606587; doi:10.1038/s41598-019-45511-6)
Supplement: Supplementary file 1 — Supplementary Material [file 41598_2019_45511_MOESM1_ESM.pdf]

Sequencing and comparative analysis of three *Chlorella* genomes provide insights into strain-specific adaptation to wastewater

Tian Wu[1,2,3#], Linzhou Li[1,2,4#], Xiaosen Jiang[1,2,3], Yong Yang[1,2,3], Yanzi Song[1,2,3], Liang Chen[5], Xun Xu[1,2,3], Yue Shen[1,2,3,6*], Ying Gu[1,2,3,6*]

1. BGI-Shenzhen, Shenzhen 518083, China
2. China National GeneBank, BGI-Shenzhen, Shenzhen 518120, China
3. Guangdong Provincial Key Laboratory of Genome Read and Write, Shenzhen 518120, China
4. School of Biology and Biological Engineering, South China University of Technology, Guangzhou 510006, China
5. Hubei Key Laboratory of Cell Homeostasis, College of Life Sciences, Wuhan University, Wuhan, Hubei 40072, China
6. Guangdong Provincial Academician Workstation of BGI Synthetic Genomics, BGI-Shenzhen, Guangdong, China

1. Supplementary Tables

Table S1. Actual piggery wastewater parameters and nutrient removal rate of *Chlorella sorokiniana* BD09 in actual piggery wastewater.

Water quality parameters	Initial measurement	Measurement after 15 days	Nutrient removal rate of BD09
COD (mg/L)	2,652	731	72%
Total N (mg/L)	321	39	88%
NH_4-N (mg/L)	271	38	86%
Total P (mg/L)	44	17	61%
pH	8.3	9.47	/

Notes:

1. Actual wastewater was diluted before *Chlorella* added.
2. Actual culture volume was 1.8 L, the initial OD_{680} of *Chlorella sorokiniana* BD09 was 1.

Table S2. Genomic and transcriptomic data statistics of three newly sequenced *Chlorella* strains.

DNA/RNA data	Sample name	Insert size	Raw read length (bp)	Raw data (Gb)	Clean data (Gb)	Sequence depth of clean data (X)
DNA	*Chlorella sorokiniana* BD09	350bp	150_150	15.5	10.56	195.6
		500bp	250_250	25.3	17.28	320
		2kb	100_100	12.4	3.79	70.2
		5kb	100_100	9.8	5.31	98.3
		10Kb	100_100	6.3	3.56	65.9
		20kb	100_100	6.4	2.66	49.3
	Total	/	/	75.7	43.16	799.3
	Chlorella sorokiniana BD08	350bp	150_150	15.5	9.49	161.9
		500bp	250_250	25.3	16.34	278.8
		2kb	100_100	12.4	6.66	113.7
		5kb	100_100	9.8	5.15	87.9
		10Kb	100_100	6.3	3.59	61.3
		20kb	100_100	6.4	2.75	46.9
	Total	/	/	75.7	43.98	750.5
	Chlorella sp. Dachan	350bp	150_150	14.6	9.72	160.9
		500bp	250_250	23.5	15.59	258.1
		2kb	100_100	10.7	4.06	67.2
		5kb	100_100	10.3	5.72	94.7
		10Kb	100_100	5.7	3.12	51.7
		20kb	100_100	6.6	2.88	47.7
	Total	/	/	71.4	41.09	680.3
RNA	*Chlorella sorokiniana* BD09	150bp	100_100	23.5	14.4	/
	Chlorella sorokiniana BD08	150bp	100_100	19.1	11.6	/
	Chlorella sp. Dachan	150bp	100_100	22.9	12.6	/

Table S3. A summary of scaffolds length distribution of our three newly sequenced genomes *Chlorella sorokiniana* BD09, *Chlorella sorokiniana* BD08 and *Chlorella sp.* Dachan in comparison with other three published *Chlorella* clade genomes.

	Chlorella sorokiniana BD08		*Chlorella sorokiniana* BD09		*Chlorella sp.* Dachan		*Chlorella variabilis* NC64A		*Chlorella sorokiniana* UTEX 1602		Micractinium *conductrix*	
**Scaffold length (bp)**	**No.**	**Size (bp)**	**No.**	**Size (bp)**	**No.**	**Size (bp)**	**No.**	**Size (bp)**	**No.**	**Size (bp)**	**No.**	**Size (bp)**
⩾ 10,000	33	57,379,143	20	53,438,449	46	58,259,782	82	45,222,670	139	59,422,181	246	60,680,316
⩾ 8,000	38	57,424,705	22	53,457,128	55	58,344,829	95	45,338,289	148	59,505,254	262	60,823,285
⩾ 5,000	43	57,455,370	23	53,463,667	72	58,454,003	134	45,602,803	155	59,552,060	283	60,962,532
⩾ 4,000	52	57,494,367	26	53,477,742	90	58,535,294	153	45,688,397	156	59,556,856	288	60,985,293
⩾ 2,000	137	57,734,923	45	53,527,384	214	58,893,416	235	45,930,001	158	59,564,266	298	61,015,730
⩾ 1,000	537	58,306,158	93	53,589,812	711	59,594,959	414	46,159,512	159	59,566,223	300	61,018,900
⩾ 800	671	58,425,778	130	53,623,002	909	59,771,270	/	/	/	/	/	/
⩾ 600	819	58,529,408	325	53,757,022	1,227	59,994,888	/	/	/	/	/	/
⩾ 500	905	58,576,441	427	53,813,149	1,385	60,082,138	/	/	/	/	/	/
⩾ 300	1,104	58,653,236	679	53,912,376	1,852	60,263,378	/	/	/	/	/	/
⩾ 200	1,242	58,686,758	920	53,971,092	2,277	60,366,751	/	/	/	/	/	/
**Total**	1,242	58,686,758	920	53,971,092	2,277	60,366,751	414	46,159,512	159	59,566,223	300	61,018,900

Table S4. The proportion of different types of repeat sequences (%) in the three newly sequenced *Chlorella* strains.

Repeat elements	***Chlorella sorokiniana* BD09**		***Chlorella sorokiniana* BD08**		***Chlorella sp.* Dachan**	
	Repeat size (bp)	% of genome	Repeat size (bp)	% of genome	Repeat size (bp)	% of genome
LTR	747,570	1.37	813,108	1.38	1,361,472	2.12
LINEs	710,610	1.31	734,058	1.24	770,850	1.2
SINEs	22,063	0.04	4,454	0	16,088	0.02
Total DNA-TEs	111,042	0.2	210,928	0.35	198,442	0.3
Satellite	1,388	0	754	0	1,993	0
Simple repeats	43,684	0.08	46,758	0.07	88,870	0.13
Other	1,970	0	1,912	0	1,217	0
Unknown	499,375	0.92	517,631	0.88	838,393	1.3
**Total**	1927537	3.56	2136707	3.63	3047886	4.76

Table S5. Number of genes with functional annotation against seven different databases in three newly sequenced *Chlorella* strains.

Type	Database	***Chlorella sorokiniana* BD09** Gene number	Percentage (%)	***Chlorella sorokiniana* BD08** Gene number	Percentage (%)	***Chlorella sp.* Dachan Dachan** Gene number	Percentage (%)
Total genes	/	9,668	100%	10,240	100%	9,821	100%
	Nr	8,550	88.44%	8,909	86.99%	8,483	86.38%
	Swissprot	6,107	63.17%	6,229	60.82%	5,934	60.42%
	KEGG	5,943	61.47%	6,112	59.68%	5,819	59.25%
Annotated	COG	4,427	45.79%	4,485	43.79%	4,318	43.97%
	TrEMBL	8,587	88.82%	8.960	87.49%	8,501	86.56%
	Interpro	7,139	73.84%	7,346	71.73%	7,012	71.40%
	GO	4,039	41.78%	4,137	40.40%	3,999	40.72%
All annotated	/	8,720	90.19%	9,128	89.13%	8,678	88.36%

Table S6. KEGG pathway enrichment of genes that were expanded in *Chlorella sorokiniana* BD09 compared to *Chlorella variabilis* NC64A (p-value < 0.05).

#	Pathway	DEGs with pathway annotation (612)	All genes with pathway annotation (5943)	P-value	Q-value	Pathway ID
1	Endocytosis	25 (4.08%)	117 (1.97%)	0.0002763611	0.02708339	ko04144
2	Plant hormone signal transduction	27 (4.41%)	138 (2.32%)	0.0007063922	0.03461322	ko04075
3	Starch and sucrose metabolism	17 (2.78%)	83 (1.4%)	0.004053814	0.13242459	ko00500
4	Stilbenoid, diarylheptanoid and gingerol biosynthesis	3 (0.49%)	6 (0.1%)	0.01712779	0.41963086	ko00945
5	Other glycan degradation	4 (0.65%)	12 (0.2%)	0.02811379	0.55103028	ko00511

Table S7. KEGG pathway enrichment of genes that were shared among *Chlorella sorokiniana* BD09, *Chlorella sorokiniana* BD08 and *Chlorella sp.* Dachan compared to *Chlorella variabilis* NC64A (p-value < 0.05).

#	Pathway	DEGs with pathway annotation (2085)	All genes with pathway annotation (17874)	P-value	Q-value	Pathway ID
1	Plant hormone signal transduction	85 (4.08%)	378 (2.11%)	1.3985e-09	1.510380e-07	ko04075
2	Endocytosis	62 (2.97%)	337 (1.89%)	0.0001758213	6.768943e-03	ko04144
3	Fatty acid elongation	15 (0.72%)	47 (0.26%)	0.0001880262	6.768943e-03	ko00062
4	Stilbenoid, diarylheptanoid and gingerol biosynthesis	7 (0.34%)	14 (0.08%)	0.0004736567	1.278873e-02	ko00945
5	Nitrogen metabolism	21 (1.01%)	99 (0.55%)	0.004563128	9.856356e-02	ko00910
6	Isoflavonoid biosynthesis	3 (0.14%)	4 (0.02%)	0.005786986	1.041657e-01	ko00943
7	Other glycan degradation	9 (0.43%)	31 (0.17%)	0.007233218	1.115982e-01	ko00511
8	Diterpenoid biosynthesis	5 (0.24%)	13 (0.07%)	0.0123529	1.586051e-01	ko00904
9	Arachidonic acid metabolism	16 (0.77%)	76 (0.43%)	0.01321709	1.586051e-01	ko00590
10	Phosphonate and phosphinate metabolism	4 (0.19%)	10 (0.06%)	0.02171426	1.956423e-01	ko00440
11	Cutin, suberine and wax biosynthesis	4 (0.19%)	10 (0.06%)	0.02171426	1.956423e-01	ko00073
12	Starch and sucrose metabolism	38 (1.82%)	234 (1.31%)	0.02173803	1.956423e-01	ko00500
13	Ether lipid metabolism	11 (0.53%)	51 (0.29%)	0.03075869	2.234067e-01	ko00565
14	Zeatin biosynthesis	4 (0.19%)	11 (0.06%)	0.03102871	2.234067e-01	ko00908
15	Lipoic acid metabolism	4 (0.19%)	11 (0.06%)	0.03102871	2.234067e-01	ko00785

Table S8. KEGG pathway enrichment of genes in the inversion region of *Chlorella sorokiniana* BD09, *Chlorella sorokiniana* BD08 and *Chlorella sp.* Dachan genomes (p-value < 0.05).

Strains	#	Pathway	DEGs with pathway annotation (202)	All genes with pathway annotation (5943)	P-value	Q-value	Pathway ID
BD09	1	DNA replication	7 (3.47%)	59 (0.99%)	0.003596674	0.2553639	ko03030
	2	Phenylalanine, tyrosine and tryptophan biosynthesis	4 (1.98%)	29 (0.49%)	0.01580532	0.4477446	ko00400
	3	Protein export	4 (1.98%)	33 (0.56%)	0.02451788	0.4477446	ko03060
	4	RNA transport	10 (4.95%)	151 (2.54%)	0.03232945	0.4477446	ko03013
	5	RNA polymerase	4 (1.98%)	36 (0.61%)	0.03263405	0.4477446	ko03020
	6	Pyrimidine metabolism	8 (3.96%)	113 (1.9%)	0.03783757	0.4477446	ko00240
	7	mRNA surveillance pathway	6 (2.97%)	77 (1.3%)	0.04611195	0.4677069	ko03015
BD08	1	Phenylalanine, tyrosine and tryptophan biosynthesis	6 (1.66%)	25 (0.41%)	0.002800962	0.1311842	ko00400
	2	DNA replication	10 (2.76%)	65 (1.06%)	0.004509143	0.1311842	ko03030
	3	Plant hormone signal transduction	17 (4.7%)	144 (2.36%)	0.004741597	0.1311842	ko04075
	4	Protein export	5 (1.38%)	29 (0.47%)	0.02595792	0.5386268	ko03060
	5	Other types of O-glycan biosynthesis	5 (1.38%)	33 (0.54%)	0.04282485	0.7108925	ko00514
Dachan	1	DNA replication	7 (4.05%)	64 (1.1%)	0.002707272	0.1597290	ko03030
	2	Protein export	4 (2.31%)	30 (0.52%)	0.01130525	0.3335049	ko03060
	3	Spliceosome	9 (5.2%)	143 (2.46%)	0.02605555	0.4425155	ko03040
	4	Base excision repair	4 (2.31%)	40 (0.69%)	0.03000105	0.4425155	ko03410
	5	Pyrimidine metabolism	7 (4.05%)	110 (1.89%)	0.04473997	0.4518410	ko00240
	6	Homologous recombination	4 (2.31%)	47 (0.81%)	0.0499743	0.4518410	ko03440

Table S9. Species list and genome versions used for comparative genomics analysis.

Species	Data Sources
Auxenochlorella protothecoides	NCBI (ASM73321v1)
Cyanidioschyzon merolae	NCBI (ASM9120v1)
Micromonas commoda	Ghent University
Chlamydomonas reinhardtii	DOE Joint Genome Institute (GCA_000002595.3)
Volvox carteri	DOE Joint Genome Institute (GCA_000143455.1)
Ostreococcus tauri	Ghent University
Picochlorum sp. SENEW3	NCBI (ASM8764v1)
Picochlorum costavermella	ORCAE (20151222)
Chlorella variabilis NC64A	DOE Joint Genome Institute (GCA_000147415.1)
Chlorella sorokiniana UTEX 1602	NCBI (ASM313072v1)
Micractinium conductrix SAG 241.80	NCBI (ASM224581v2)

Table S10. The composition of BG11 medium.

Compound	Concentration (mg/liter)
$NaNO_3$	1,500
K_2HPO_4	40
$MgSO_4.7H_2O$	75
$CaCl_2.2H_2O$	36
Citric acid	6
Ammonium ferric citrate green	6
$EDTANa_2$	1
Na_2CO_3	20
Trace metal solution A5	1 ml/liter

The detailed information of A5:

Compound	Concentration (mg/liter)
H_3BO_3	2,860
$MnCl_2.4H_2O$	1,810
$ZnSO_4.7H_2O$	222
$Na_2MoO_4.2H_2O$	390
$CuSO_4.5H_2O$	79
$Co(NO_3)_2.6H_2O$	494

Adjust pH to 7.1 with 1M NaOH or HCl.

2. Supplementary Figures

a

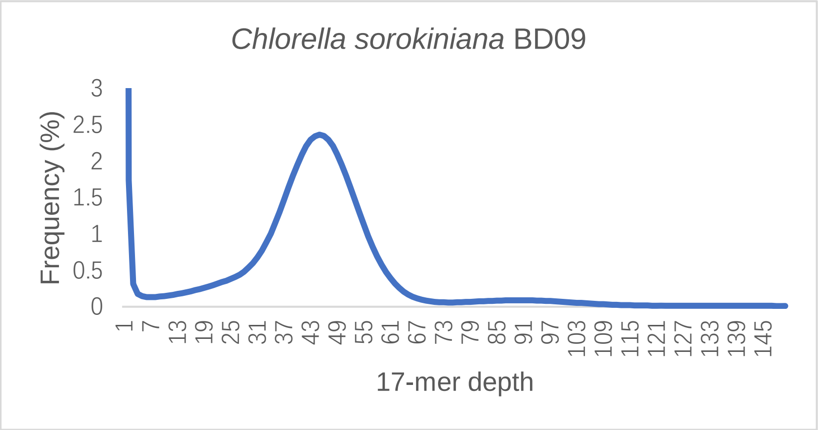

b

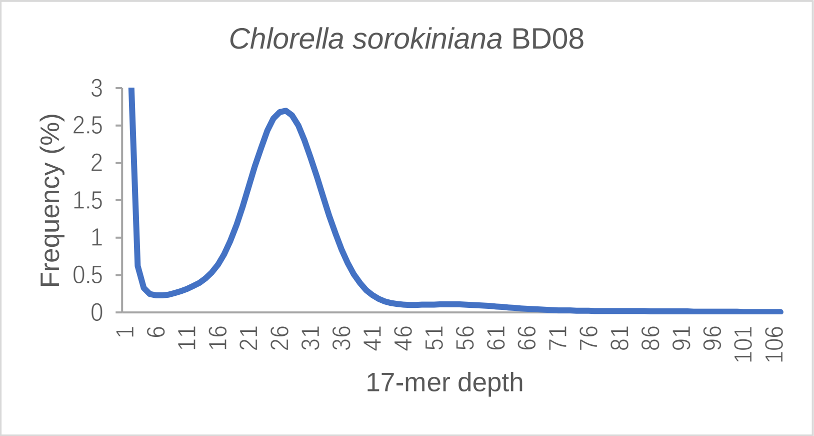

c

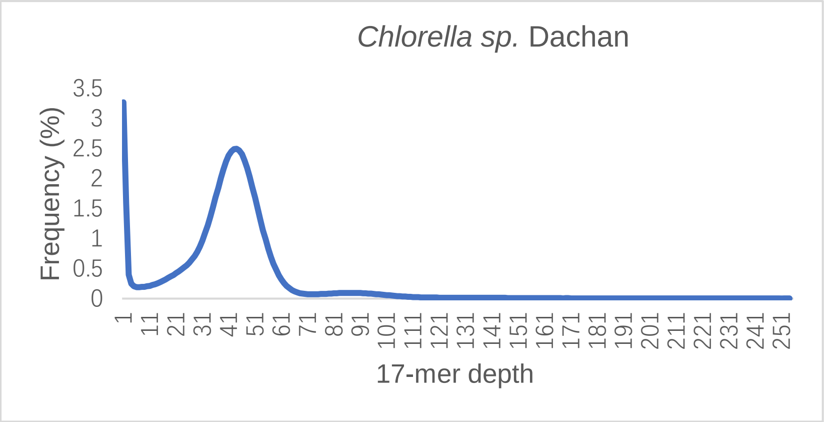

Figure S1. 17-kmer estimation of genome sizes among three newly sequenced *Chlorella* strains. The x-axis was depth (X); the y-axis was K-mer frequency. a) The genome size of *Chlorella sorokiniana* BD09 was estimated to be 55.1 Mb based on reads from short insert size library. b) The genome size of *Chlorella sorokiniana* BD08 was estimated to be 56.5 Mb based on reads from short insert size library. c) The genome size of *Chlorella sp.* Dachan was estimated to be 60.4 Mb based on reads from short insert size library.

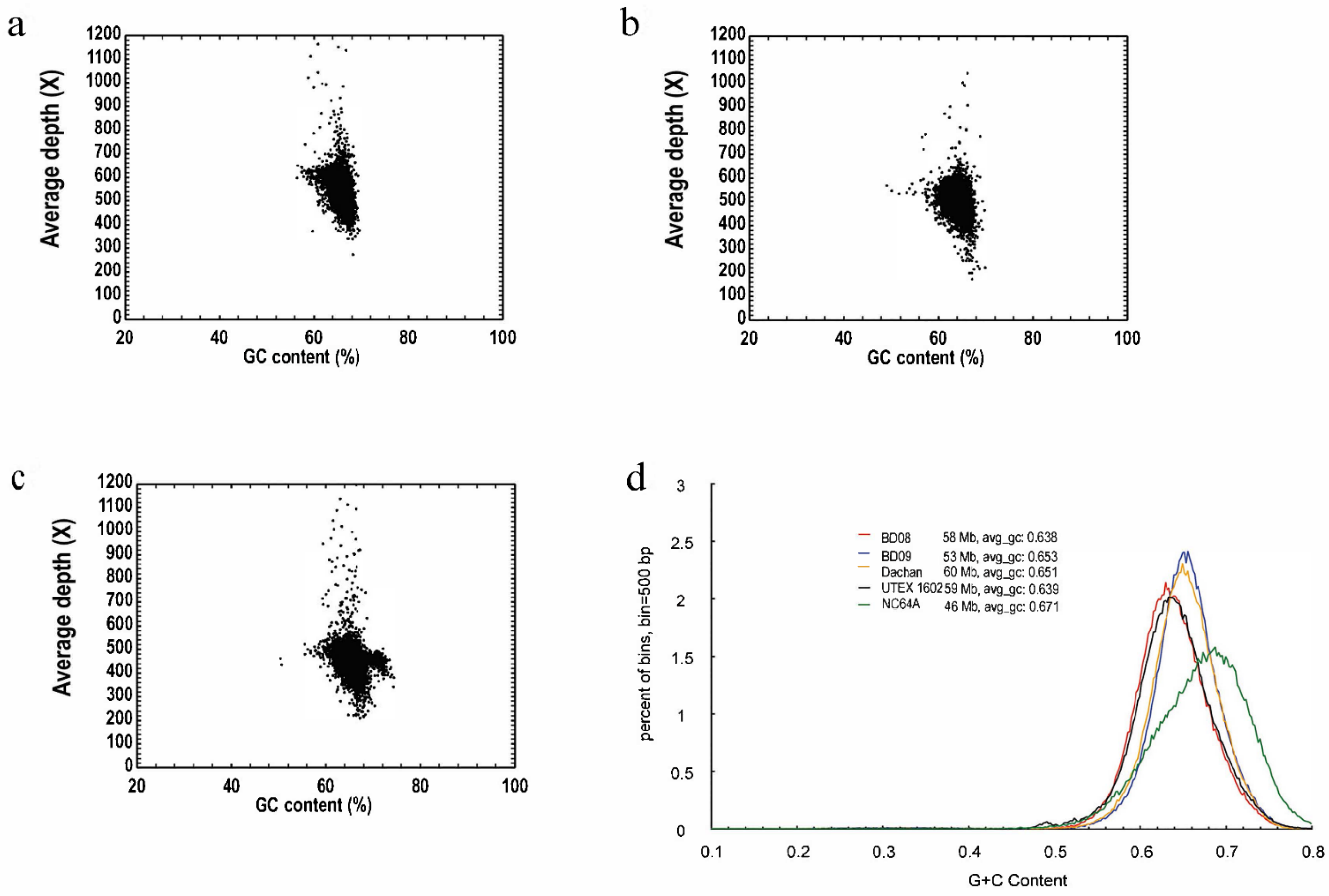

Figure S2. GC content analysis of three newly sequenced genomes (a, b, c) and comparison of the GC contents across five *Chlorella* genomes (d). a) GC content analysis of *Chlorella sorokiniana* BD09; b) GC content analysis of *Chlorella sorokiniana* BD08; c) GC content analysis of *Chlorella sp.* Dachan. In the figure d, the x-axis was GC content and the y-axis indicated the proportion of the bin number divided by the total windows. We used 500 bp bins (with 250 bp overlap) sliding along the genome. The GC content of three genomes was 65.3% (BD09), 63.8% (BD08) and 65.1% (Dachan) respectively, which was similar to *Chlorella sorokiniana* UETX 1602 (63.9%). All four genomes were GC rich that slightly lower than *Chlorella variabilis* NC64A (67.1%).

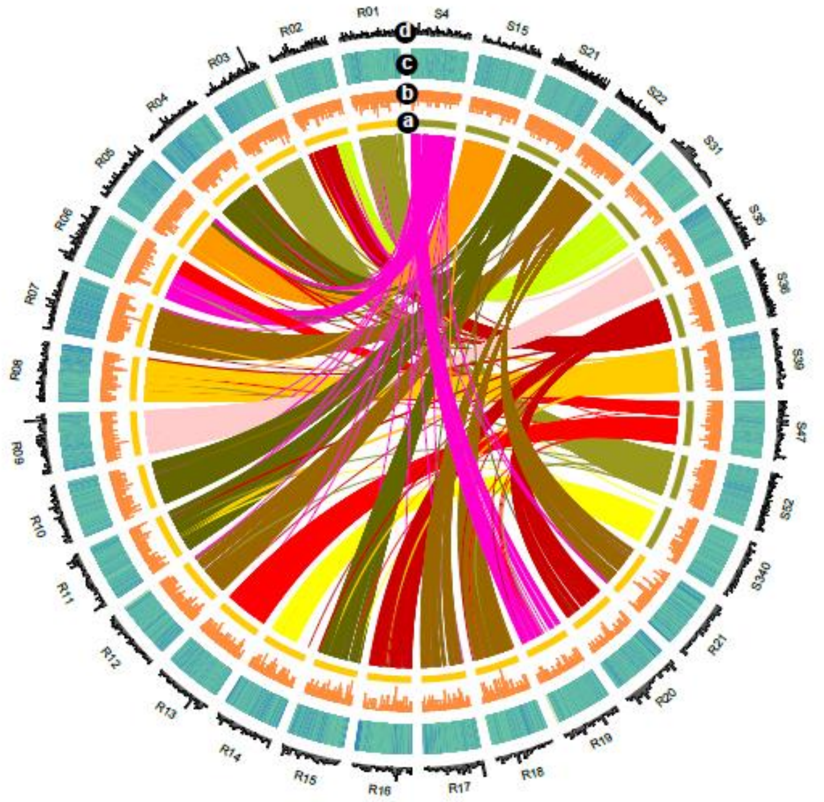

Figure S3. Circos visualization of collinearity between *Chlorella sorokiniana* BD08 (S) and *Chlorella sorokiniana* UTEX 1602 (R), only scaffolds with length >1 Mb were used. Different layers denoted: a) collinear regions between the two strains connected by colored lines, b) gene density, c) GC content, and d) TE density.

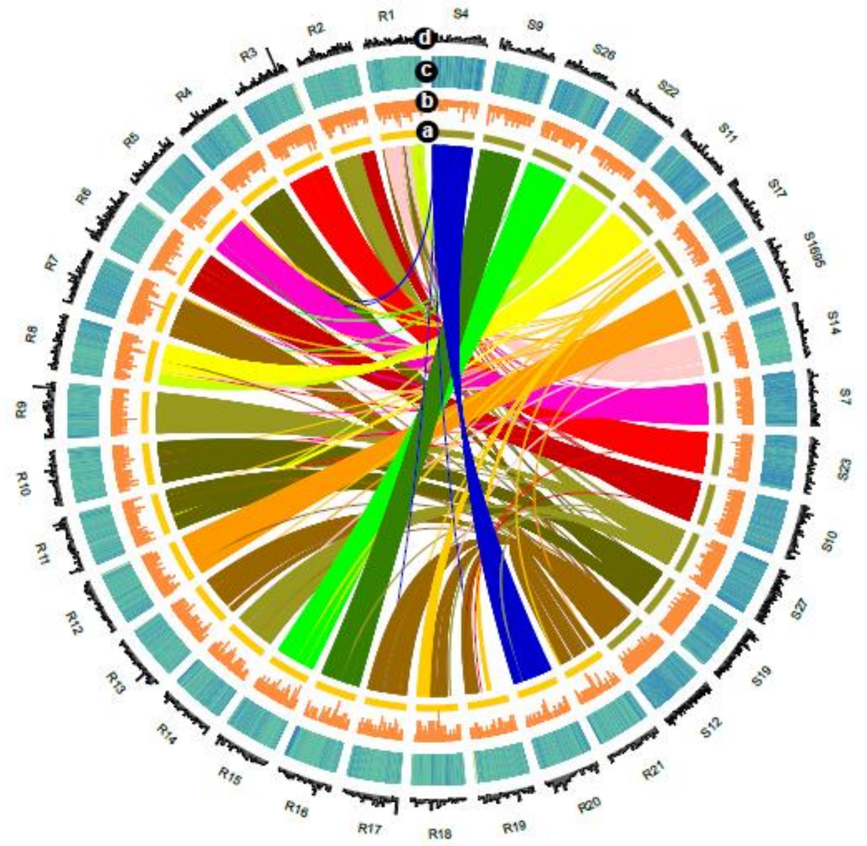

Figure S4. Circos visualization of collinearity between *Chlorella sorokiniana* BD09 (S) and *Chlorella sorokiniana* UTEX 1602 (R), only scaffolds with length >1 Mb were used. Different layers denoted: a) collinear regions between the two strains connected by colored lines, b) gene density, c) GC content, and d) TE density.

a

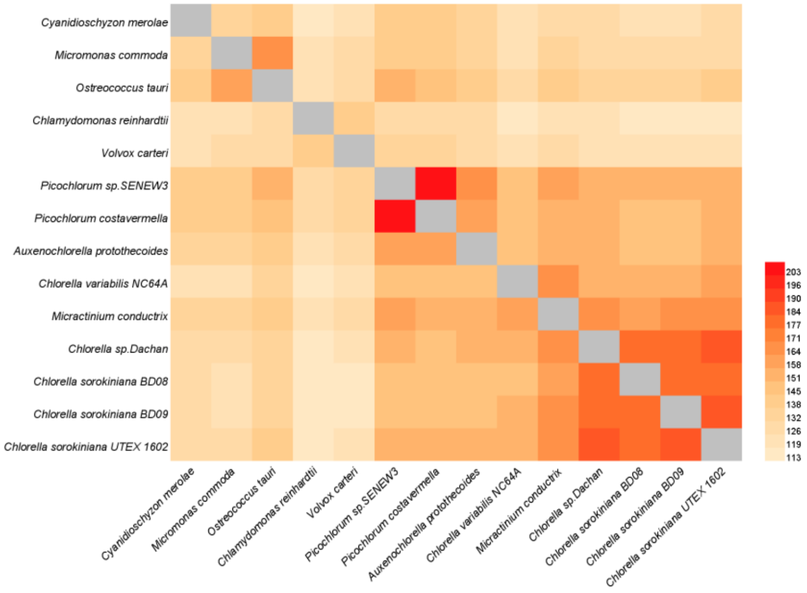

b

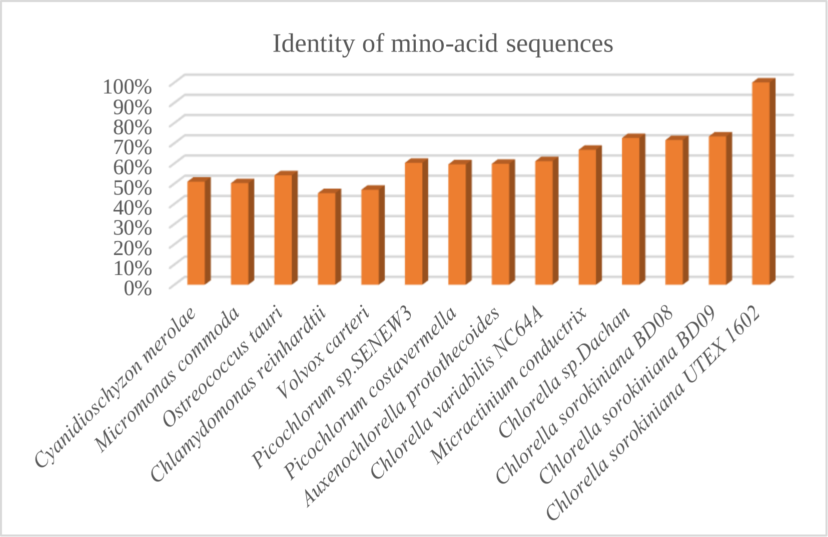

Figure S5. Identity comparation of amino-acid sequences of 14 algal genomes. a) A heatmap of average blast bit score of 14 genomes using the protein coding genes (e-value ≤ 1e-10). The self-blasted results were represented by the color grey. b) A bar chart of amino-acid similarity statistics of 13 genomes in comparation with *Chlorella sorokiniana* UTEX 1602.
